# Supplementary material for: Metabolic analysis of amino acids and vitamin B6 pathways in lymphoma survivors with cancer related chronic fatigue
Source: PLoS One. 2020 Jan 10;15(1):e0227384. doi: 10.1371/journal.pone.0227384 (PMC6953873; doi:10.1371/journal.pone.0227384)
Supplement: S1 Table — (DOCX) [file pone.0227384.s001.docx]

**S1 Table:** Amino acid concentrations in lymphoma survivors

|  | All patients | | | Male patients | | | Female patients | | |
| --- | --- | --- | --- | --- | --- | --- | --- | --- | --- |
| Category I  µM, mean (SD^a^) | With CF^b^  n=77 | Without CF  n=167 | P | With CF  n=44 | Without CF  n=109 | P | With CF  n=33 | Without CF  n=58 | P |
| Glycine* | 292.5 | 291.4 | 0.92 | 262.1 | 272.0 | 0.27 | 332.9 | 327.9 | 0.80 |
|  | (80.7) | (72.8) |  | (48.2) | (50.5) |  | (96.9) | (92.4) |  |
| Serine* | 137.6 | 139.3 | 0.56 | 132.0 | 137.2 | 0.17 | 144.9 | 143.3 | 0.73 |
|  | (23.8) | (20.6) |  | (22.8) | (20.2) |  | (23.4) | (21.0) |  |
| Cysteine | 311.6 | 312.0 | 0.91 | 313.3 | 311.2 | 0.70 | 309.3 | 313.5 | 0.57 |
|  | (31.9) | (30.9) |  | (32.2) | (28.2) |  | (31.7) | (35.8) |  |
| Threonine | 133.5 | 140.3 | 0.08 | 135.6 | 141.2 | 0.20 | 130.7 | 138.7 | 0.26 |
|  | (29.0) | (27.3) |  | (26.0) | (24.0) |  | (32.8) | (32.6) |  |
| Alanine* | 425.1 | 439.4 | 0.25 | 444.0 | 453.0 | 0.59 | 400.0 | 413.9 | 0.41 |
|  | (95.1) | (88.3) |  | (101.1) | (92.0) |  | (81.3) | (75.1) |  |
| Category II  µM, mean (SD) |  |  |  |  |  |  |  |  |  |
| Lysine | 195.5 | 203.6 | 0.07 | 199.0 | 206.1 | 0.20 | 190.8 | 198.9 | 0.20 |
|  | (32.6) | (29.5) |  | (33.4) | (30.3) |  | (31.3) | (27.7) |  |
| Leucine | 139.4 | 142.8 | 0.36 | 149.1 | 153.3 | 0.32 | 126.5 | 123.0 | 0.47 |
|  | (27.4) | (26.1) |  | (24.6) | (22.9) |  | (25.8) | (19.6) |  |
| Phenylalanine | 74.3 | 72.9 | 0.24 | 75.9 | 74.3 | 0.26 | 72.1 | 70.2 | 0.36 |
|  | (10.1) | (8.1) |  | (8.6) | (7.7) |  | (11.5) | (8.1) |  |
| Tyrosine | 70.9 | 72.7 | 0.30 | 74.8 | 75.1 | 0.89 | 65.7 | 68.2 | 0.34 |
|  | (13.6) | (12.3) |  | (12.5) | (12.2) |  | (13.3) | (11.2) |  |
| Isoleucine | 72.6 | 74.1 | 0.51 | 79.0 | 80.0 | 0.69 | 64.0 | 63.0 | 0.73 |
|  | (17.4) | (15.8) |  | (16.3) | (14.4) |  | (15.3) | (11.9) |  |
| Tryptophan | 73.4 | 77.6 | 0.01 | 76.9 | 80.1 | 0.10 | 68.8 | 72.9 | 0.09 |
|  | (12.9) | (11.0) |  | (11.5) | (11.0) |  | (13.4) | (9.6) |  |
| Category III  µM, mean (SD) |  |  |  |  |  |  |  |  |  |
| Valine | 271.1 | 276.2 | 0.40 | 284.7 | 291.5 | 0.35 | 252.9 | 247.6 | 0.54 |
|  | (46.9) | (43.2) |  | (44.1) | (38.5) |  | (44.8) | (36.7) |  |
| Methionine | 28.9 | 29.9 | 0.14 | 30.4 | 30.9 | 0.49 | 26.9 | 27.8 | 0.33 |
|  | (5.3) | (4.5) |  | (4.9) | (4.4) |  | (5.2) | (4.0) |  |
| Asparagine/  Aspartic acid | 83.3 | 84.1 | 0.57 | 84.8 | 84.6 | 0.91 | 81.2 | 83.1 | 0.47 |
|  | (11.7) | (9.8) |  | (10.2) | (9.3) |  | (13.3) | (10.6) |  |
| Histidine* | 81.2 | 83.2 | 0.14 | 82.0 | 83.5 | 0.38 | 80.2 | 82.6 | 0.35 |
|  | (11.4) | (9.0) |  | (9.1) | (9.8) |  | (14.0) | (7.3) |  |
| Proline* | 197.4 | 206.9 | 0.30 | 211.0 | 222.2 | 0.37 | 179.4 | 178.1 | 0.91 |
|  | (59.5) | (68.5) |  | (57.0) | (74.8) |  | (58.7) | (42.1) |  |
| Glutamine/  Glutamic acid* | 633.9 | 642.0 | 0.37 | 644.5 | 646.5 | 0.85 | 619.6 | 633.6 | 0.39 |
|  | (72.8) | (62.1) |  | (64.6) | (57.5) |  | (81.4) | (69.5) |  |

^a^Standard deviation; ^b^ Chronic fatigue *Data with non-normal distribution, Mann-Whitney U test reported.
