# Supplementary material for: Metabolic analysis of amino acids and vitamin B6 pathways in lymphoma survivors with cancer related chronic fatigue
Source: PLoS One. 2020 Jan 10;15(1):e0227384. doi: 10.1371/journal.pone.0227384 (PMC6953873; doi:10.1371/journal.pone.0227384)
Supplement: S2 Table — (DOCX) [file pone.0227384.s002.docx]

**S2 Table:** Arginine and tricarboxylic acid cycle metabolites in lymphoma survivors

|  | All patients | | | Male patients | | | Female patients | | |
| --- | --- | --- | --- | --- | --- | --- | --- | --- | --- |
| Arginine related metabolites  µM, mean (SD^a^) | With CF^b^  n=77 | Without CF  n=167 | P | With CF  n=44 | Without CF  n=109 | P | With CF  n=33 | Without CF  n=58 | P |
| Arginine* | 103.4  (18.2) | 107.1  (19.8) | 0.17 | 104.4  (18.3) | 106.9  (20.8) | 0.49 | 102.0  (18.4) | 107.4  (17.9) | 0.17 |
| Homoarginine* | 1.84  (0.68) | 1.93  (0.77) | 0.35 | 2.02  (0.75) | 1.95  (0.70) | 0.58 | 1.60  (0.50) | 1.91  (0.98) | 0.07 |
| Asymmetric  Dimethylarginine | 0.55  (0.10) | 0.54  (0.10) | 0.83 | 0.54  (0.11) | 0.55  (0.10) | 0.64 | 0.55  (0.10) | 0.53  (0.09) | 0.29 |
| Symmetric  Dimethylarginine* | 0.68  (0.18) | 0.67  (0.16) | 0.72 | 0.67  (0.16) | 0.66  (0.14) | 0.60 | 0.60  (0.21) | 0.70  (0.18) | 0.88 |
| Ornithine | 84.5  (19.1) | 86.7  (19.6) | 0.40 | 88.5  (17.1) | 87.9  (18.5) | 0.85 | 79.0  (20.5) | 84.4  (21.5) | 0.25 |
| Urea* | 6.0  (1.7) | 6.2  (1.9) | 0.45 | 6.3  (1.4) | 6.2  (1.5) | 0.68 | 5.6  (2.0) | 6.2  (2.5) | 0.25 |
| Tricarboxylic acid cycle metabolites,  µM, mean (SD) |  |  |  |  |  |  |  |  |  |
| α-ketoglutaric acid* | 9.2  (2.3) | 8.5  (1.9) | 0.01 | 9.12  (2.4) | 8.4  (1.8) | 0.10 | 9.3  (2.1) | 8.5  (2.0) | 0.08 |

^a^Standard deviation; ^b^ Chronic fatigue *Data with non-normal distribution, Mann-Whitney U test reported.
