# Supplementary material for: Metabolic analysis of amino acids and vitamin B6 pathways in lymphoma survivors with cancer related chronic fatigue
Source: PLoS One. 2020 Jan 10;15(1):e0227384. doi: 10.1371/journal.pone.0227384 (PMC6953873; doi:10.1371/journal.pone.0227384)
Supplement: S3 Table — (DOCX) [file pone.0227384.s003.docx]

**S3 Table:** Metabolites of kynurenine pathway in lymphoma survivors

|  | All patients | | | Male patients | | | Female patients | | |
| --- | --- | --- | --- | --- | --- | --- | --- | --- | --- |
| Metabolite  nM, mean (SD^a^) | With CF^b^  n=77 | Without CF  n=167 | P | With CF  n=44 | Without CF  n=109 | P | With CF  n=33 | Without CF  n=58 | P |
| Kynurenine (in µM)* | 2.04 | 2.00 | 0.71 | 2.09 | 2.00 | 0.38 | 1.96 | 2.00 | 0.79 |
|  | (0.72) | (0.52) |  | (0.64) | (0.47) |  | (0.81) | (0.60) |  |
| 3-Hydroxykynurenine* | 63.2 | 56.1 | 0.07 | 64.9 | 54.5 | 0.04 | 60.9 | 59.2 | 0.79 |
|  | (36.2) | (24.1) |  | (39.6) | (21.1) |  | (31.4) | (28.8) |  |
| Kynurenic acid* | 70.1 | 67.0 | 0.51 | 71.6 | 69.4 | 0.73 | 68.2 | 62.6 | 0.44 |
|  | (33.4) | (34.7) |  | (29.8) | (37.1) |  | (38.1) | (29.3) |  |
| Xanthurenic acid* | 19.8 | 19.8 | 0.97 | 21.0 | 20.3 | 0.71 | 18.3 | 18.7 | 0.87 |
|  | (9.4) | (9.8) |  | (9.0) | (9.2) |  | (9.8) | (10.9) |  |
| Anthranilic acid* | 20.4 | 19.1 | 0.38 | 18.8 | 18.9 | 0.95 | 22.4 | 19.3 | 0.26 |
|  | (12.1) | (10.1) |  | (8.4) | (9.9) |  | (15.6) | (10.5) |  |
| 3-Hydroxyanthranilic acid | 52.5 | 57.1 | 0.09 | 58.0 | 59.6 | 0.64 | 45.3 | 52.5 | 0.07 |
|  | (20.6) | (18.7) |  | (22.1) | (18.2) |  | (16.0) | (18.9) |  |
| Picolinic acid* | 35.7 | 33.4 | 0.27 | 38.5 | 34.5 | 0.12 | 32.0 | 31.3 | 0.84 |
|  | (20.4) | (11.3) |  | (20.4) | (10.5) |  | (20.0) | (12.7) |  |
| Quinolinic acid* | 620.8 | 552.2 | 0.25 | 591.1 | 525.1 | 0.20 | 660.5 | 602.9 | 0.66 |
|  | (615.1) | (305.6) |  | (368.9) | (248.3) |  | (844.3) | (388.8) |  |
| Nicotinamide | 200.2 | 201.5 | 0.93 | 206.1 | 205.1 | 0.96 | 192.2 | 194.6 | 0.89 |
|  | (102.9) | (105.9) |  | (119.6) | (119.5) |  | (76.1) | (74.2) |  |
| N1-methylnicotinamide* | 137.1 | 139.5 | 0.83 | 138.8 | 140.9 | 0.90 | 134.8 | 136.7 | 0.89 |
|  | (74.6) | (83.7) |  | (78.1) | (93.7) |  | (70.8) | (61.3) |  |
| Kynurenine/Tryptophan ratio* | 0.029 | 0.026 | 0.06 | 0.028 | 0.026 | 0.09 | 0.031 | 0.028 | 0.21 |
|  | (0.016) | (0.009) |  | (0.009) | (0.008) |  | (0.023) | (0.010) |  |

^a^Standard deviation; ^b^ Chronic fatigue *Data with non-normal distribution, Mann-Whitney U test reported.
