# Supplementary material for: Metabolic analysis of amino acids and vitamin B6 pathways in lymphoma survivors with cancer related chronic fatigue
Source: PLoS One. 2020 Jan 10;15(1):e0227384. doi: 10.1371/journal.pone.0227384 (PMC6953873; doi:10.1371/journal.pone.0227384)
Supplement: S4 Table — (DOCX) [file pone.0227384.s004.docx]

**S4 Table:** Neopterin and metabolites of vitamin B6 in lymphoma survivors

|  | All patients | | | Male patients | | | Female patients | | |
| --- | --- | --- | --- | --- | --- | --- | --- | --- | --- |
| Metabolite  nM, mean (SD^a^) | With CF^b^  n=77 | Without CF  n=167 | P | With CF  n=44 | Without CF  n=109 | P | With CF  n=33 | Without CF  n=58 | P |
| Neopterin* | 27.1 | 23.1 | 0.07 | 28.1 | 23.1 | 0.07 | 25.9 | 23.0 | 0.35 |
|  | (17.8) | (9.8) |  | (16.1) | (10.1) |  | (20.0) | (9.3) |  |
| Pyridoxal 5’-phosphate* | 46.9 | 57.9 | 0.16 | 43.0 | 60.0 | 0.11 | 52.1 | 53.9 | 0.87 |
|  | (44.3) | (61.1) |  | (24.6) | (68.1) |  | (61.7) | (45.3) |  |
| Pyridoxal* | 22.8 | 30.8 | 0.59 | 19.1 | 35.9 | 0.48 | 27.8 | 21.4 | 0.45 |
|  | (39.7) | (127.1) |  | (11.4) | (156.8) |  | (59.3) | (19.6) |  |
| 4-Pyridoxic acid* | 37.8 | 44.9 | 0.68 | 34.7 | 49.4 | 0.59 | 41.8 | 36.4 | 0.64 |
|  | (38.7) | (147.0) |  | (24.9) | (177.9) |  | (51.9) | (53.1) |  |
| PAr index^b^* | 0.638 | 0.421 | 0.006 | 0.630 | 0.499 | 0.03 | 0.650 | 0.477 | 0.04 |
|  | (0.42) | (0.28) |  | (0.41) | (0.31) |  | (0.43) | (0.21) |  |

^a^Standard deviation; ^b^ PAr index calculated as the ratio of 4-pyridoxic acid divided by sum of concentrations of pyridoxal 5'-phosphate and pyridoxal;^c^ Chronic fatigue *Data with non-normal distribution, Mann-Whitney U test reported.
