# Supplementary material for: Metabolic analysis of amino acids and vitamin B6 pathways in lymphoma survivors with cancer related chronic fatigue
Source: PLoS One. 2020 Jan 10;15(1):e0227384. doi: 10.1371/journal.pone.0227384 (PMC6953873; doi:10.1371/journal.pone.0227384)
Supplement: S5 Table — (DOCX) [file pone.0227384.s005.docx]

**S5 Table:** Logistic regression analyses with chronic fatigue as dependent variable

|  | Univariate analysis | | | Multivariable | | |
| --- | --- | --- | --- | --- | --- | --- |
|  | OR^e^ | 95% CI^r^ | P | OR | 95% CI | P |
| Female sex (reference male) | 1.41 | 0.81-2.45 | .22 | 0.49 | 0.18-1.32 | .49 |
| Age at survey (years) | 0.99 | 0.97-1.01 | .46 | 0.98 | 0.95-1.01 | .23 |
| Mediastinal radiotherapy | 1.89 | 1.09-3.29 | .02 | 1.36 | 0.66-2.81 | .40 |
| Neuroticism score | 1.62 | 1.38-1.91 | <.001 | 1.50 | 1.21-1.87 | <.001 |
| Impact of event scale score | 1.04 | 1.02-1.06 | <.001 | 1.02 | 0.99-1.05 | .14 |
| HADS^a^-A | 1.22 | 1.13-1.32 | <.001 |  |  |  |
| HADS-D | 1.36 | 1.23-1.50 | <.001 |  |  |  |
| Body mass index ≥ 30 kg/m^2^ | 2.50 | 1.20-5.22 | .02 | 3.11 | 1.22-7.97 | .02 |
| VO_2_^b^ peak (l/min) | 0.59 | 0.38-0.92 | .02 | 0.48 | 0.22-1.05 | .07 |
|  |  |  |  |  |  |  |
| Serum IL-6^c^ detectable | 2.25 | 1.26-4.00 | .006 |  |  |  |
| Par^d^ index | 3.56 | 1.55-8.22 | .003 | 3.62 | 1.05-12.46 | 0.04 |

Variables with p ≤0.10 in univariate analysis are shown in addition to age and sex.^a^ Hospital anxiety and depression scale; ^b^ Volume of oxygen; ^c^ Interleukin 6; ^d^  PAr index calculated as the ratio of 4-pyridoxic acid divided by sum of concentrations of pyridoxal 5'-phosphate and pyridoxal; ^e^ Odds Ratio; ^f^ Confidence interval
