# Supplementary material for: Metabolic analysis of amino acids and vitamin B6 pathways in lymphoma survivors with cancer related chronic fatigue
Source: PLoS One. 2020 Jan 10;15(1):e0227384. doi: 10.1371/journal.pone.0227384 (PMC6953873; doi:10.1371/journal.pone.0227384)
Supplement: S1 Fig — Correlations of neopterin (A) and kynurenine/tryptophan ratio (B) with PAr index (ratio of 4-pyridoxic acid divided by sum of concentrations of pyridoxal 5'-phosphate and pyridoxal) in survivors. (DOCX) [file pone.0227384.s006.docx]

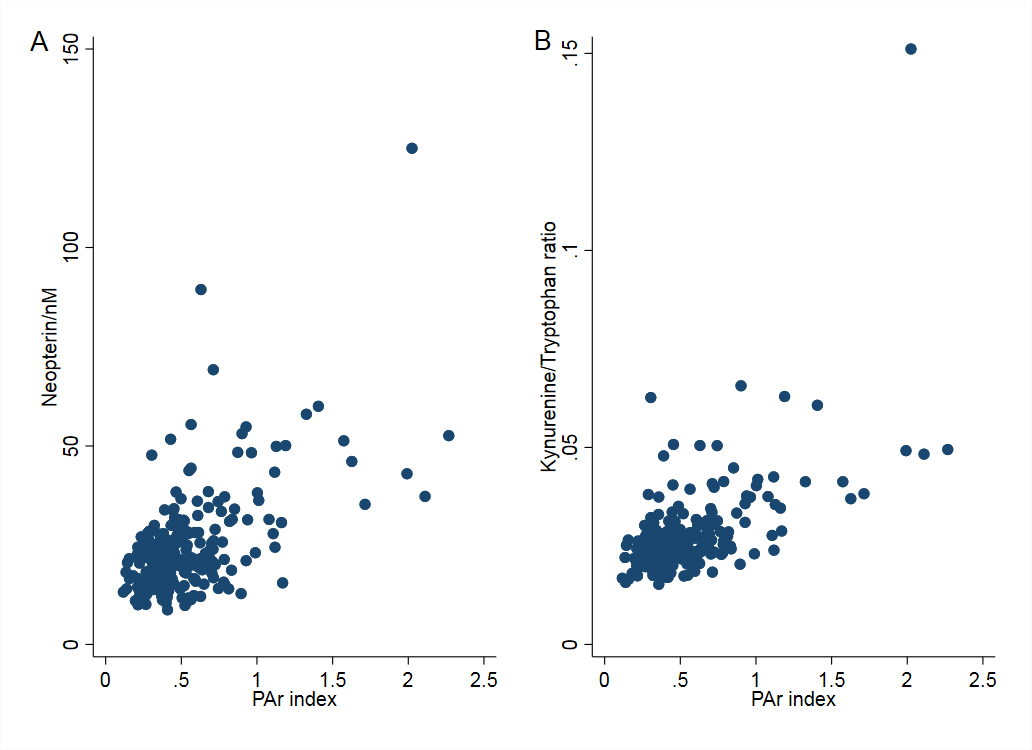


**S1 Fig:** Correlations of Neopterin (A) and Kynurenine/Tryptophan ratio (B) with PAr index (ratio of 4-pyridoxic acid divided by sum of concentrations of pyridoxal 5'-phosphate and pyridoxal) in survivors.
